# Two Gastroenteritis Outbreaks Caused by GII Noroviruses: Host Susceptibility and HBGA Phenotypes

**DOI:** 10.1371/journal.pone.0058605

**Published:** 2013-03-05

**Authors:** Miao Jin, Yaqing He, Huiying Li, Pengwei Huang, Weiming Zhong, Hong Yang, Hailong Zhang, Ming Tan, Zhao-jun Duan

**Affiliations:** 1 National Institute for Viral Disease Control and Prevention, China CDC, Beijing, China; 2 Divisions of Infectious Diseases, Cincinnati Children’s Hospital Medical Center, Cincinnati, Ohio, United States of America; 3 Department of Pediatrics, University of Cincinnati College of Medicine, Cincinnati, Ohio, United States of America; 4 Major Infectious Disease Control Key Laboratory, Shenzhen Center for Disease Control and Prevention, Shenzhen, China; The Australian National University, Australia

## Abstract

Noroviruses (NoVs) cause epidemic acute gastroenteritis, in which histo-blood group antigens (HBGAs) may play an important role in the host susceptibility. To further explore this issue, two outbreaks of acute gastroenteritis caused by a GII.4 and a GII.3 NoV, respectively, in China in 2009 were studied. Stool and saliva samples from symptomatic patients and water samples from the outbreak facilities were collected. RT-PCR showed that 23 out of 33 (GII.4 outbreak) and 12 out of 13 (GII.3outbreak) stool samples were NoV positive. For the GII.4 outbreak the NoV sequences of stool and water samples were from an identical GII.4 strain, while the same GII.3 NoV sequences were found in five stool samples from the GII.3 outbreak. The HBGA phenotypes (A, B, Le^a^, Le^b^, Le^x^, and Le^y^) of all saliva samples were determined, which revealed both secretors and nonsecretors in the symptomatic groups of the two outbreaks. In the GII.3 outbreak, type O individuals appeared less susceptible, while the type A may be more at risk of infection. However, No preference of HBGAs was observed in the GII.4 outbreak. The observation that nonsecretors were infected in both outbreaks differed from the previous results that nonsecretors are resistant to these two GII NoVs.

## Introduction

Noroviruses (NoVs) are a group of round-structured RNA viruses, constituting the *Norovirus* genus in the family *Caliciviridae*. These non-enveloped viruses consist of an outer protein capsid that encapsulates a single stranded, positive sense RNA genome of ∼7.5 kb [1]. NoVs have emerged as a leading cause of viral acute gastroenteritis in both children and adults and are responsible for numerous outbreaks worldwide[2], [3], [4], [5]. Genetically NoVs are divided into five genogroups (G) [1], in which GI, GII, and GIV NoVs infect humans, while GIII and GV NoVs infect bovine and murine, respectively. Vast majority of human NoV epidemics are caused by GI and GII NoVs that are further divided into at least 8 and 17 genotypes, respectively[6].GII.4 and GII.3 NoVs are the first and second predominant genotypes in causing most human NoV diseases. NoV outbreaks often occur in closed and semi-closed facilities, such as cruise ships, battle ships, nursing homes, hospitals, schools, and military camps[5], .

Increasing data showed that human histo-blood group antigens (HBGAs) play an important role in the host susceptibility of NoVs (reviewed in [9], ). HBGAs are complex carbohydrates distributing abundantly on red bold cells and mucosal surface of gastro-intestinal tracts. They also exist as free oligosaccharides in body fluids including saliva, intestinal content and milk. Early studies revealed that recombinant NoV capsids, the virus like particles (VLPs),and the isolated protruding (P) domain of NoV capsid bound to HBGAs in a strain-specific manner [15], [16], [17], [18], [19], [20], [21]. Direct evidence of such interaction came from crystallography studies of NoV P domains in complex with the HBGA ligands[22], [23], [24], [25], [26], [27], confirmed by structure-guided mutagenesis [28], [29],[30]. These structural data have greatly advanced our understanding of the complex interactions between the diverse NoVs and the polymorphic HBGAs.

The association between the susceptibility of NoV infection and the HBGA phenotypes of hosts was showed by human challenge studies and investigations of outbreaks of acute gastroenteritis caused by NoVs. Human volunteers challenged with Norwalk virus (NV, GI.1) showed a host range matching well with the binding profile of NV VLPs to saliva samples, in which type O and A secretors are at high risk, type B secretor at low risk, while nonsecretors are resistant to NV infection [31], [32], [33].Similarly, another human challenge with a GII.4 NoV showed infection with illness to secretor but not nonsecretor individuals, consistent with the binding pattern of the challenges train [34]. In addition, a number of investigations on gastroenteritis outbreaks caused by NoVs supported the association of NoV infection and illness with the HBGA phenotypes of hosts[35], [36], [37], [38], [39], [40], [41]. However, discrepancies were also reported. For example, challenge of human volunteers with Snow Mountain virus (SMV, GII.2) did not reveal a preference to a particular HBGA phenotype or blood type, although SMV VLPs bind only to type B saliva [42]. Similar results were also reported based investigations of some NoV outbreaks[43], [44], although concerns were raised to the methodology of those outbreak investigations[45], [46].

In summary of the available data, while the specific interactions between recombinant NoV VLPs/P domains and HBGAs have been clearly shown and an association of NoV susceptibility with the HBGA phenotypes of hosts appeared to be a general rule, exceptions and variations were observed. To further explore this issue, we studied two outbreaks of acute gastroenteritis caused by a Den Haag variant of GII.4 NoV and a GII.3 NoV, respectively. The outcomes apparently differed from the those reported previously [37], in which both secretor and nonsecretor individuals were found susceptible to the two NoVs. Our results raised new questions on possible alterations of the complex interactions of the NoVs with the HBGAs and their clinical consequences.

## Subjects, Materials and Methods

### Ethics statement

The study was approved by the Institutional Review Board (IRB) at China CDC for human subject protection. A written informed consent was obtained from the symptomatic patients and the asymptomatic individuals for all collected samples including stool specimen and saliva samples.

### Outbreak data and sample collection

Two outbreaks of acute gastroenteritis occurred in Shenzhen city of southern China in 2009. The first outbreak, referred as GII.4 outbreak, occurred in big industrial company, in which 258 people were sick with acute gastroenteritis. 33 stool and55 saliva samples from symptomatic patients and 43 water samples from the company water reservoirs were collected. 23 saliva samples from asymptomatic individuals were also collected for comparison. The second outbreak, designated as GII.3 outbreak, occurred in a village in the suburb of same city, in which 35people were reportedly ill with acute gastroenteritis. 13 stool and 19 saliva samples from symptomatic patients were collected. In addition, 20saliva samples from asymptomatic individuals of the same village were collected as controls. For both outbreaks symptomatic patients was defined as presence of clinical symptoms: watery diarrhea and/or vomiting. Description of symptoms was obtained through a questionnaire given to each of all participants in the study.

Stool samples were collected through a conventional protocol [47]. Only on stool sample was collected from each selected symptomatic patient, typically 12 to 24 hours after onset disease. Water samples (1000 mL each) were taken from the company’s reservoir at a position 30 centimeters below water surface by a sterile, stainless steel ladle or were collected directly from a tap. Water samples were held by sterile glass flasks. 1000 mL water samples were concentrated to 0.5 mL by the membrane adsorption-elution method and 200ìL were directly used for viral RNA extraction [48]. Saliva samples were collected by asking each individual to spit saliva into a 15 mL plastic tube and stored at -20°C. Saliva samples were boiled and diluted before use for HBGA phenotyping.

### RNA extraction and real-time PCR

A 10% stool suspension was prepared by mixing 0.1 gram stool with 1.0 ml phosphate-buffered saline (PBS, pH 7.2). Viral RNA was extracted from the clarified stool suspensions using the QiaAmp Viral RNA Mini Kit (Qiagen, Hilden, Germany) according to the manufacturer’s protocol. Viral RNA was examined for GI and GII NoVs in a duplex format using the QuantiTect Probe RT-PCR kit (Qiagen, Hilden, Germany) on a 7500Real-time PCR platform (Applied Biosystem). The final reaction mix of 25 ìL consisted of 0.4 µM of each of the four primers[Cog1F, Cog1R, Cog2F, Cog2R, [49]], and 0.2ìM of each TaqMan Probe Ring 1a, Ring1b and Ring 2 [49]. Cycling conditions included a step of reverse transcription for 30 min at 50 Cand a denaturation for 15 min at 95 C, followed by 40 cycles of amplification, in which each cycle contains a denaturation for 15 seconds at 95 C and a combination of annealing and extension for 1 min at 60 C.

### Conventional Reverse transcription (RT)-PCR

Several stool and water samples were further analyzed for detection of NoVs by conventional RT-PCR targeting the capsid region for NoV genotyping. The RT-PCR conditions and the used primers (CoG2F, G2-SKR, G1-SKF and G1-SKR) were described elsewhere [50].

### HBGA Phenotyping of Saliva samples

The HBGA phenotypes of A, B, Le^a^, Le^b^, Le^x^, and Le^y^ antigens of the saliva samples were determined by EIA assays using the corresponding monoclonal antibodies against individual HBGAs as described previously [15], [37]. Briefly, boiled and diluted saliva (1∶1000) was coated on microtiter plates. After blocking with 5% nonfat milk, monoclonal antibodies against individual antigens (Signet Laboratories Inc., Dedham, MA)(1∶100)were added. Then the corresponding secondary antibody-horseradishperoxidase (HRP) conjugates (Immunology Consultants Laboratory Inc., Newberg, OR) were added. The signal intensities were displayed by adding HRPsubstrate reagents(optEIA, BD Bioscience, San Diego, CA). Four well-characterized saliva samples containing all interested HBGAs were included in each plate as internal controls. The cut-off of a positive signal wasOD_450_ = 0.1. Among all 117 saliva samples4 (3.4%) showed no detectable signals of any HBGA. Since reason for the loss of the HBGA signals remain unknown these 4 samples were omitted from the study.

### Amplification of the capsid protein-encoding gene for *in vitro* expression

The P domain-encoding cDNA sequences were amplified by PCR using a sense primer(ACGCGTCGACTCGAATTCTCAAGAACTAAACCATTTACTGT) and an anti-sense primer (GCATGCGGCCGCTTAGCAAAAGCAATCGCCACGGCAATCGCATAAAGCACGCCTACGCCCCGT). This resulted in the P domain-encoding cDNA with sequences coding a cystein-containing tag (CDCRGDCFC) at the 3′ end, potentially promoting the P domain to form P particle[20], [51]. The resulting PCR product was cloned into the expression vector pGEX-4T-1 (GE Healthcare Life Sciences) between BamHI and NotI sites. Recombinant P protein was expressed in *E.coli* as described previously[20]. VLP was also attempted to produce using the established protocol as described elsewhere [16], [52].

### DNA Sequencing and phylogenetic analysis

For DNA sequencing NoV PCR products were cloned into the pGEM-T vector (Promega, Medison, WI).The nucleotide sequences of the inserted NoV cDNA were determined using the Big-Dye terminator cycle sequencing kit and the ABI Prism 310 Genetic Analyzer (Applied Biosystems Inc., Foster City, CA).The nucleotide sequences generated in this study were deposited in GenBank under accession number JX282191.The resulting NoV sequences were analyzed using CLUSTAL X (Version 1.83) followed by phylogenetic analysis using MEGA version 4.1. The statistical significance of the inferred phylogenies was estimated using bootstrap analysis with 1,000 pseudo replicate data sets.

### Statistical Analysis

This was calculated by the software GraphPadInStat (version 3.06, GraphPad Software, Inc.). The associations of the HBGA phenotypes with the symptomatic infection were analyzed by Chi-square test, the association of the secretor status with the symptomatic infection by Fisher’s exact test, and the distribution difference of an individual blood type between the symptomatic and asymptomatic groups by Student’s t-test based on their signal measurements of the EIAs, respectively.

## Results

### Description of the two NoV outbreaks

The first outbreak of acute gastroenteritis occurred in a large company in southern China from September 21 to October 3, 2009. 258 people were reported sick with acute gastroenteritis. The first case was reported on September 21 followed by many cases in progress towards the peak of the outbreak on September 26 with63 cases in a single day. The most common symptoms were watery diarrhea (83%), followed by abdominal pain (40%), abdominal distension (35%), nausea (33%), malaise (28%), vomiting (19%), and fever (3%). The second outbreak occurred in a village of the same area between September 29 and October 10, 2009, in which 35 people were sick with acute gastroenteritis.

**Figure 1:**
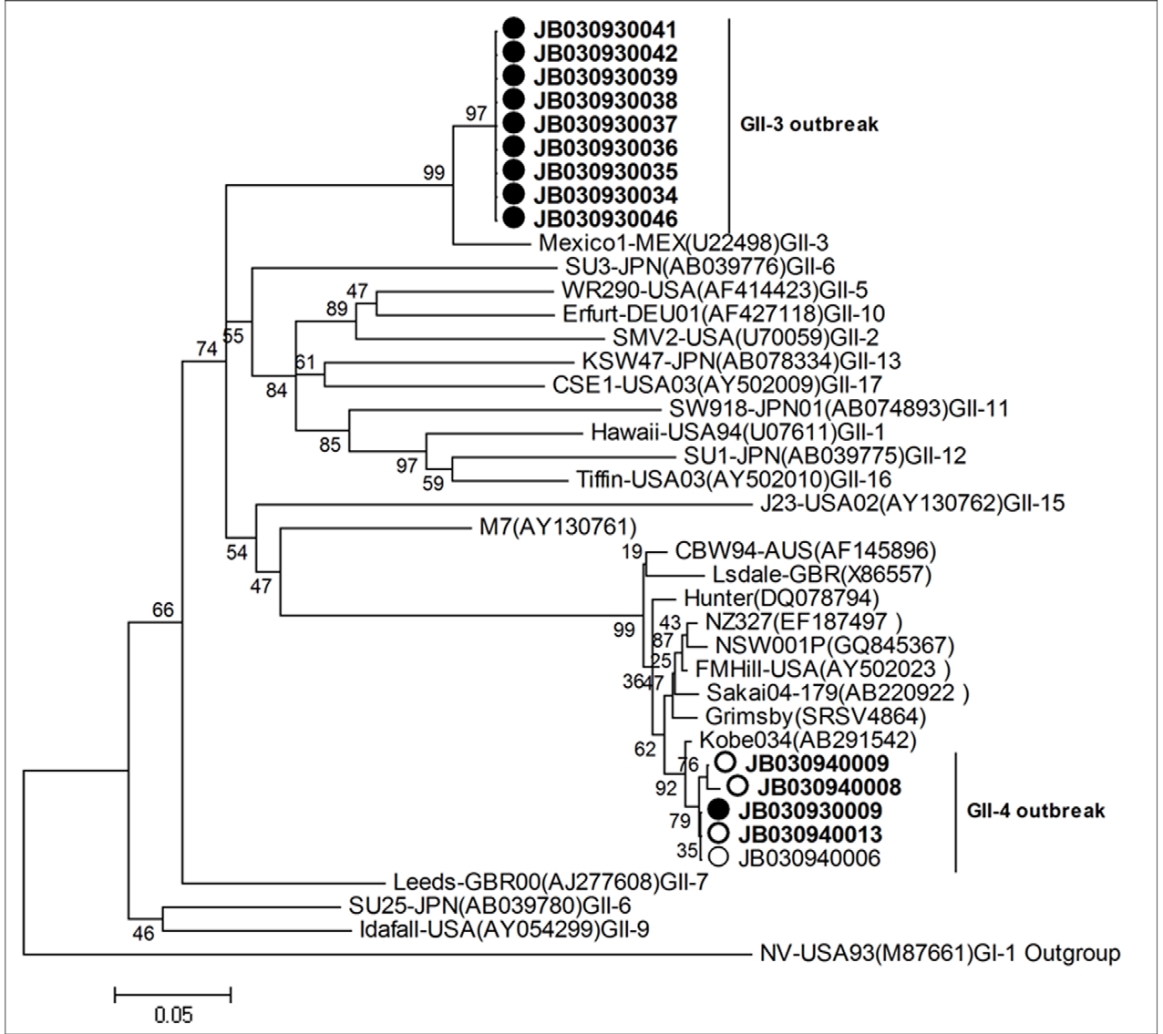
Phylogenetic tree of the two outbreak noroviruses based on the nucleotide sequences of the P domain from the outbreaks. The sequences from stool samples were indicated by solid circles, while those from water samples were indicated by hollow circles. The tree was constructed using the neighbor-joining method and the numbers on each branch indicated bootstrap values. Branch lengths are proportional to the evolutionary distance between sequences, and the distance scale, in nucleotide substitutions per position, is indicated. The reference strains are depicted by italic with indications of GenBank accession numbers.

Real-time PCR revealed that 23 out of 33 and 12 out of 13 stool samples from the symptomatic patients of the first and the second outbreaks, respectively were NoV positive. In addition, 8 out of 43 water samples of the first outbreak were NoV positive. The NoV sequences of the first outbreak were amplified by conventional RT-PCR from three stool and four water samples and sequenced. All seven resulting sequences revealed an identical GII.4 NoV, suggesting that the contaminated water reservoirs may be the infection source. Phylogenetic analysis showed this NoV as a Den Haag variant of GII.4 NoV (Figure 1). Therefore, this outbreak was designated as GII.4 outbreak. On the other hand, All PCR products from five stool samples collected from symptomatic patients of the second outbreak revealed the same NoV sequences, which represents a variant of the mexico-1 (MEX)cluster of GII.3 NoVs(Figure 1). Thus, the second outbreak was designated as GII.3 outbreak. All water samples for the GII.3 outbreak were NoV negative, thus the infection source of the outbreak remained unknown. The stool and water samples were also tested for common enteropathogenic bacteria (Shigella, Cholera, typhoid and paratyphoid fever) and other viral pathogens (sapovirus, rotavirus, astrovirus and adenovirus) by conventional bacteria culture procedures and RT-PCR, respectively, which were all negative.

### Distribution of the HBGA phenotypes

**Figure 2:**
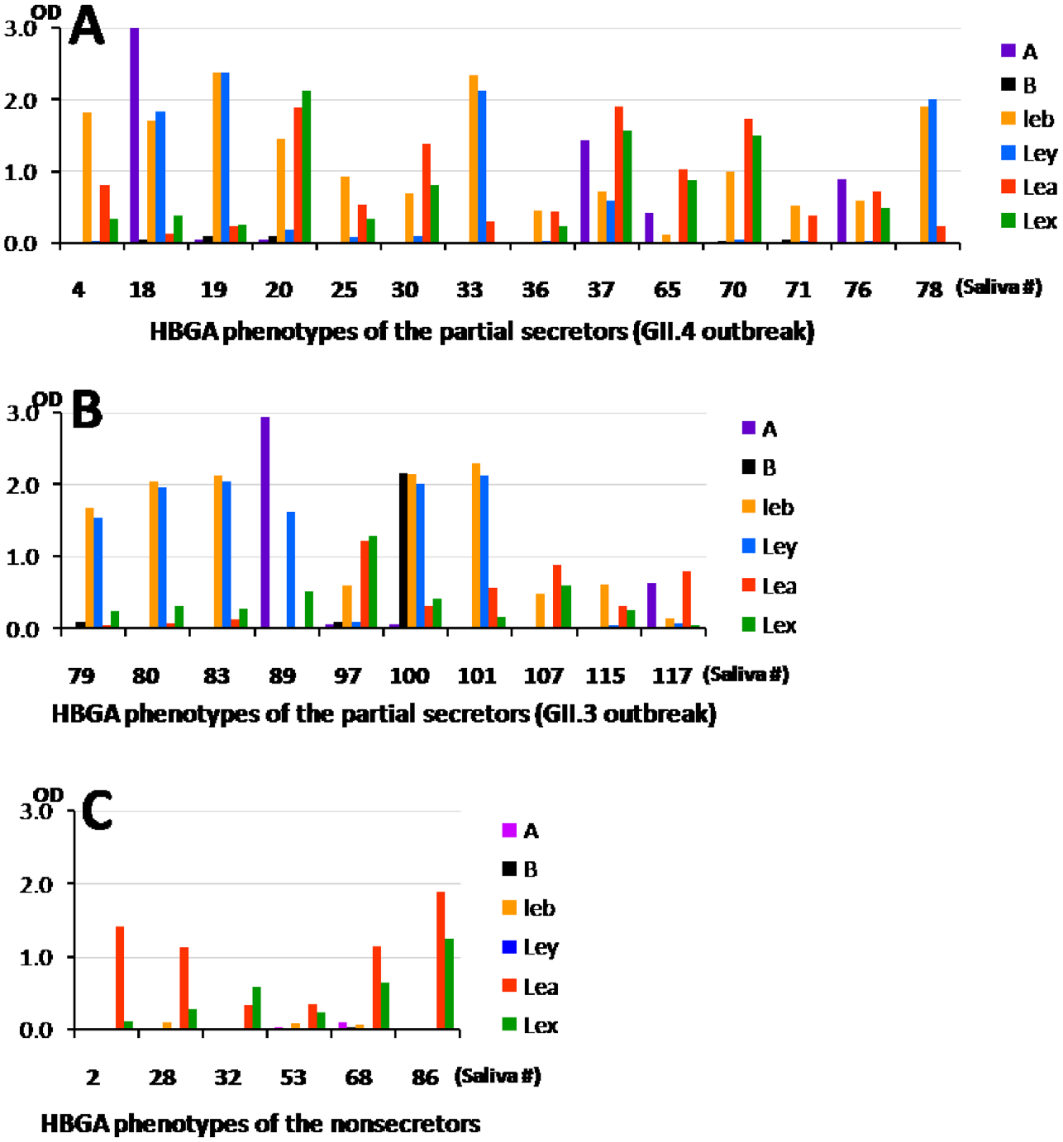
Determination of nonsecretor statuses by histo-blood group antigen (HBGA) phenotyping. (A and B) The HBGA (A, B, Le^a^, Le^b^Le^x^, and Le^y^) phenotypes of 14 symptomatic (A) and 10 asymptomatic (B) individuals from the two outbreaks who were Le^a^ and/or Le^x^as well as A/B/Le^b^/Le^y^ positive. These individuals were referred as partial nonsecretors or partial secretors. (C) 5 (#2, 28, 32, 53 and 68) and 1(#86) individuals in the GII.4 and GII.3 outbreaks, respectively, showed strong Le^a^ and/or Le^x^ signals but no detectable or marginal signals of A, B, Le^b^, and Le^y^ antigens. These individuals were referred as nonsecretors. Y-axis, signal intensity (optical density, OD)of HBGAs detected by the ELISA.X-axis, saliva samples.

The HBGA phenotypes of A, B, Le^a^, Le^b^, Le^x^, and Le^y^ antigens of each saliva sample were determined by EIAs (Table 1). Typical distributions of ABO blood types were seen among the asymptomatic individuals compared with those of the Chinese populations(http://www.bloodbook.com/world-abo.html). The Le^b^ and/or Le^y^ positive individuals were commonly seen among secretor individuals, which is typical[37]. We noted that 14 symptomatic (19%) and 10 asymptomatic (24%) secretors (Le^b^/Le^y^ and/or A/B positive) were also positive for Le^a^ and/or Le^x^ (Figure 2A and B). These individuals are referred as partial secretors or partial nonsecretors and they were sorted into secretors group. 5 (7%) and 1 (3%) nonsecretor individuals were identified in the GII.4 and GII.3 outbreaks, respectively, whose saliva showed strong Le^a^ and/or Le^x^ signals but no detectable or only marginal signals of Le^b^, Le^y^, A and B antigens (Figure 2C and Table1).

**Table 1:** Distribution of the histo-blood group antigen phenotypes among individuals with or without symptoms of acute gastroenteritis.

	GII.4 outbreak			GII.3 outbreak		
HBGAs	Symptomatic	Asymptomatic	Total	Symptomatic	Asymptomatic	Total
	#(%), n = 53	# (%), n = 21	# (%), n = 74	# (%), n = 19	# (%), n = 20	# (%), n = 39
ABO phenotypes1:						
O	24(45)	11 (52)	35 (47)	8 (42)	14 (70)	22 (56)
A	20 (38)	8 (38)	28 (38)	4 (21)	2 (10)	6 (15)
B	7 (13)	2 (10)	9 (12)	4(21)	4 (20)	8 (21)
A/B	2 (4)	0 (0)	2(3)	3 (16)	0 (0)	3 (8)
Lewis phenotypes:						
Le^b^	45(85)	19 (90)	64 (88)	15 (79)	18 (90)	33 (85)
Le^y^	44(83)	15(71)	59 (73)	17 (89)	15 (75)	32 (82)
Le^a^	14(26)	7 (33)	21(30)	4 (21)	5 (25)	9 (23)
Le^x^	14 (26)	5 (24)	19 (4)	6 (32)	4 (20)	10 (25)
Secretor status:						
Positive	49 (92)	20 (95)	69 (93)	18 (95)	20 (100)	38 (97)
Negative	4(8)	1 (5)	5 (7)	1(5)	0(0)	1(3)

### HBGA phenotypes and Symptomatic Infection

The distribution rates of the HBGA phenotypes, including the secretor statuses, between the symptomatic and asymptomatic groups turned out to be very similar in the GII.4 outbreak (Table 1). No clear preference of HBGAs between the two groups was seen. For the GII.3 outbreak, type O secretors appeared less susceptible as 70% of the asymptomatic individuals had this blood type vs. 42% in the symptomatic group(*P* = 0.079). In contrast, the type A individuals could be at higher risk of infection because 21% of the symptomatic patients had this blood type, while only 10% of the asymptomatic individuals had it (*P* = 0.407). However, both differences were not statistically significant, probably due to the low number of sample pools. To our surprise, 4 and 1 nonsecretors individuals were found in the symptomatic groups of the GII.4 and GII.3 outbreak, respectively, suggesting that nonsecretor individuals were also susceptible to these two particular GII NoVs.

### Analysis of the HBGA-binding domain of the GII.4 outbreak virus

**Figure 3.Comparison:**
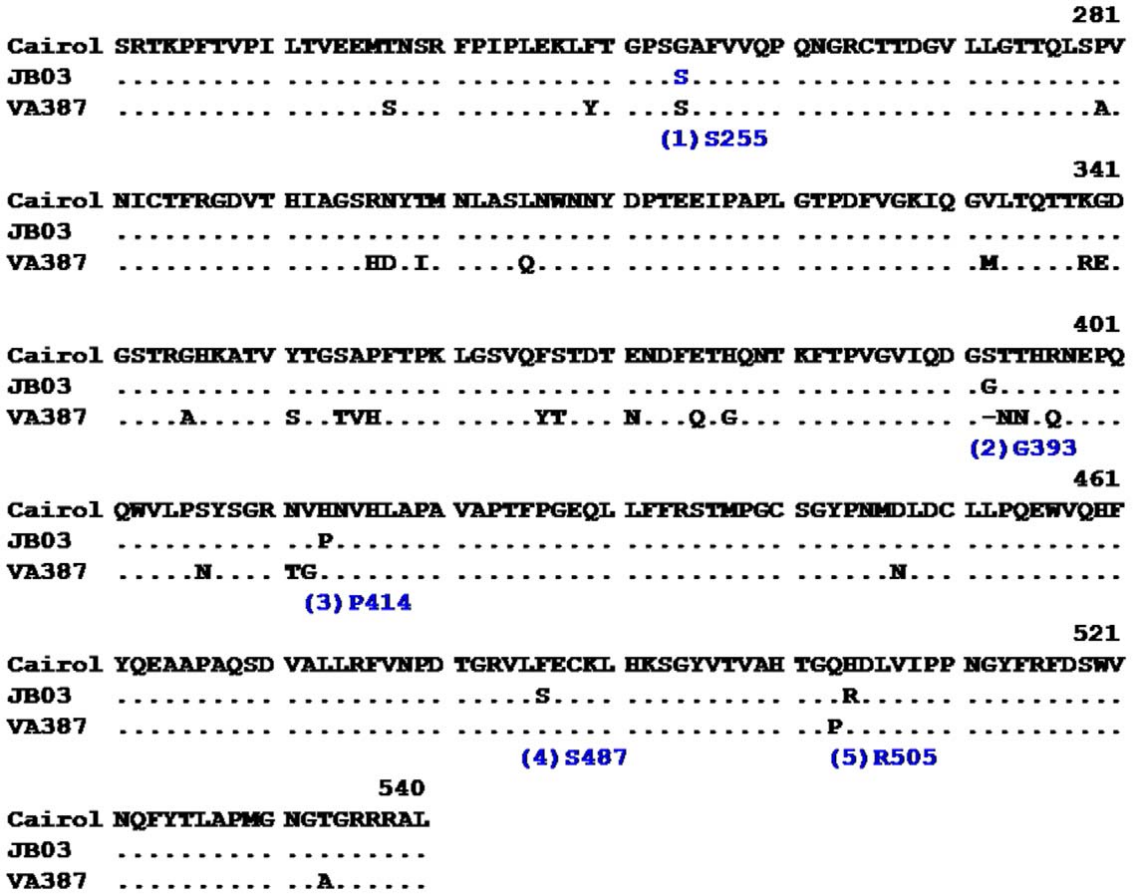
of the P domain sequences of the GII.4 outbreak virus (JB03) with Cairo1 (EU876892), another Den Haag variant that binds to nonsecretor saliva and antigens, and VA387 (AY038600), a GII.4 NoV with known structure of HBGA-binding sites. The three conserved components of the HBGA-binding site are indicated by red empty rectangles (dashed lines), while the five amino acids of JB03 that differ from Cairo1 are marked by blue rectangles and indicated by numbers in parentheses according to their sequence order. The amino acids with their sequence position were also indicated.

We first attempted to produce recombinant P particles and VLPs of the GII.4 outbreak NoV (JB03) using the established protocols for HBGA-binding profile determination [16], [20], but was not successful (see discussion). To explore the possible binding property of the GII.4 outbreak virus we compared the deduced amino acid sequences of the P domain, the HBGA-binding domain, of JB03with those of other two well-characterized GII.4 NoVs. They were VA387, a 1997 strain with known crystal structure of the HBGA binding sites[23], andCairo1, a2006 Den Haag variant that binds Le^x^ and sialyl Le^x^antigens and saliva of nonsecretors [30](Figure 3).

**Figure 4:**
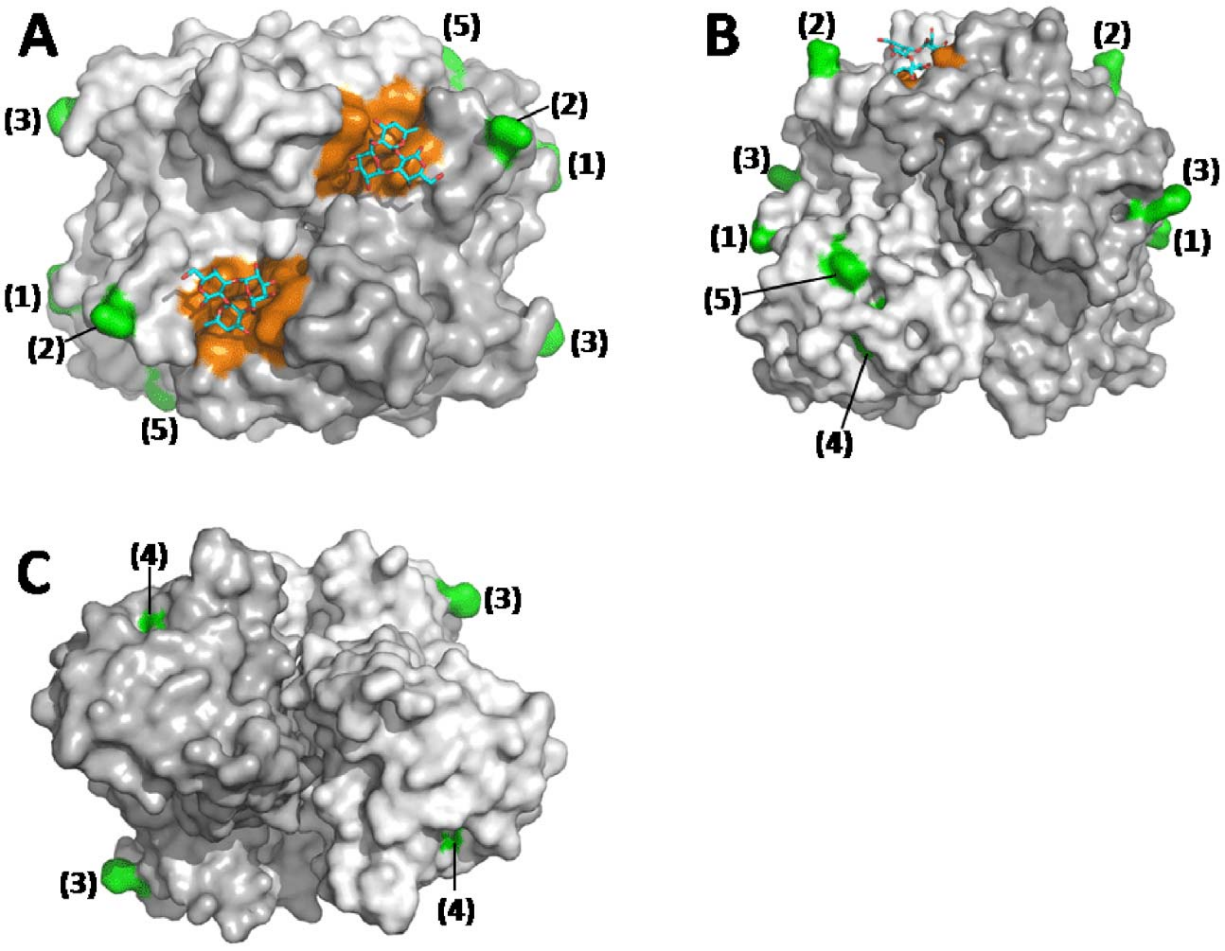
The crystal structures of the P domain dimer (surface model) of GII.4 VA387 norovirus viewed at top (A), side (B) and bottom (C) directions, respectively. The two P domain protomers are shown in light and dark grey, respectively, with indications of two symmetric histo-blood group antigen (HBGA)-binding sites (orange) on the top surface of the P dimer. The type B-trisaccarides are indicated in stick representation (cyan-red-blue) on each binding site. The locations of the five amino acids of JB03 (green)that differ from Cairo1 norovirus are indicated by numbers in parentheses according to their sequence order. All these five amino acids are away from the conserved HBGA-binding sites.

Remarkably, JB03 P domain shares 98.7% amino acid identity with that of Cairo1, differing in only five residues (Figure 3). Analysis based on the crystal structure of VA387 P dimer revealed that all five residues are located on the P dimer surface, among which four (S255, P414, S487 and R505) are located at the sides or bottom of the P dimer, far away from the conserved HBGA binding sites of NoVs (Figure 4). Thus, these four amino acid mutations are unlikely to change the binding profile of Cairo1. Interestingly, the remaining mutation(G393S) is located near a site that can affect the binding specificity [27], [28], [30], [53]. However, since both glycine and serine are tiny, structurally similar amino acids, aS393G mutation may not affect the HBGA binding profile of Cairo1 significantly, In other words, the JB03 may share the same or similar binding profile with Caro1 that bind to nonsecretor saliva and antigens [30].

## Discussion

NoVs cause worldwide epidemics of acute gastroenteritis. Due to their natures of wide spread and low infection dose NoVs are difficult to control and prevent. Thus, further understanding the epidemiology and host susceptibility of NoVs will help our combat against these viruses. We reported here an investigation of two outbreaks of acute gastroenteritis caused by a GII.4 and a GII.3 NoV, respectively. We found that both secretor and nonsecretor individuals were susceptible to the two viruses, which differed from the results reported previously on the same two NoV genotypes that infected only secretors[37]. Our new findings raised questions again on the debating possibility that NoVs may change their target populations through altering their HBGA binding properties.

The two outbreaks were showed to be caused by a single GII.4 and a GII.3 NoV, respectively, while the involvement of other common enteric bacterial and viral pathogens was apparently excluded. We amplified the full-length VP1-encoding gene of the GII.4 virus (JB03) for construction of the recombinant VLPs and P particles. However, both productions were unsuccessful as we failed to obtain expected VLPs/P particle reagents for determination of the HBGA-binding profile of the outbreak virus. This was not unusual because similar scenario occurred in our past experience in producing NoV VLPs and P particles, which could be VP1 or P domain sequence-dependent. Fortunately, a very similar Den Haag variant of GII.4 NoV, Cairo1 (EU876892), was well characterized recently [30], which revealed binding ability to both secretor and nonsecretor saliva samples and oligosaccharides of Le^x^ and sialyl Le^x^ antigens. Cairo 1 and JB03 P domains share high homology (98.7%) in their amino acid sequences and the only five different amino acids are located away from the conserved HBGA-binding sites of NoVs (Figure 3 and Figure 4). Based on our current understanding of the NoV-HBGA interaction, the GII.4 outbreak NoV (JB03) may share the same or similar HBGA-binding profile with that of Cairo 1. Thus, the infection outcomes of the GII.4 outbreak caused by the JB03 NoV could possibly represent the clinical outcomes of the Cairo 1 strain.

In addition to their consistent bindings to secretor HBGAs, interactions of recombinant VLPs of some other GII.4 NoVs to nonsecretor saliva and antigens have also been reported. These included1) Dijon171 (AF472623)[30], [54], a US95/96 (1996) variant, 2) 2002(2002a)[53], a variant of the 2002 cluster, 3) Valencia/2004/Es[35], and 4)Cairo4 (EU876884)[30], an Osaka (2007) variant. A case of infection of a nonsecretor individual by the GII.4 Valencia/2004/Es strain was reported in 2009 [35]. Our findings provided a further example of such possibility that nonsecretor may be susceptible to some GII.4 strains. Continue study on the susceptibility of the nonsecretors to other GII.4 strains is of significance.

The involvement of the common enteric bacterial and other viral pathogens, including Shigella, Cholera, typhoid, paratyphoid fever, sapovirus, rotavirus, astrovirus and adenovirus have been examined and excluded. However, the possibility of contribution by other untested enteric pathogens to the outcomes of outbreaks cannot be excluded. Therefore, future study in this context would be necessary to accumulate more data as further proofs. On the other hand, unlike GII.4 NoVs, whose epidemiology and host susceptibility have been better studied, those of GII.3 NoVs remained poorly understood. A recent study showed that four GII.3 VLPs representing different GII.3 clusters (I, II, III, and NC) bound to Le^x^ antigen, a nonsecretor phenotype [55], suggesting that GII.3 NoVs may be able to infect nonsecretors. Thus our observation serves as a further piece of evidence for this possibility. Production of VLPs and P particles of the GII.3 outbreak virus is ongoing in our laboratory, which would further help our understanding of this outbreak NoV.
